# Mendelian randomization and FinnGen analysis of the causal relationship between 473 gut microbiota species and chronic sinusitis

**DOI:** 10.1016/j.bjorl.2025.101711

**Published:** 2025-09-25

**Authors:** Chenguang Zhang, Yicong Wang, Chenghao Hu, Bin Guo, Huwei Jiang, Chunlong Zhao, Yuwen Wang

**Affiliations:** a Graduate School of Qinghai University, Cheng bei District, Xining, Qinghai, People's Republic of China; b Affiliated Hospital of Qinghai University, Department of Otolaryngology, Cheng xi District, Xining, Qinghai, People's Republic of China; c Affiliated Hospital of Qinghai University, Department of Gastrointestinal Oncology, Cheng xi District, Xining, Qinghai, People's Republic of China; d Affiliated Hospital of Qinghai University, Department of Hepatobiliary and Splenic Surgery, Cheng xi District, Xining, Qinghai, People's Republic of China; e School of Medicine, Zhejiang University, Xi hu District, Hangzhou, Zhejiang, People's Republic of China

**Keywords:** Mendelian randomization, Chronic sinusitis, Gut microbiota, Precision medicine, Genome-Wide Association Study (GWAS)

## Abstract

**Objective:**

To investigate the causal associations between Gut Microbiota (GM) and Chronic Sinusitis (CRS) using Mendelian Randomization (MR).

**Methods:**

Genome-Wide Association Study (GWAS) summary statistics for 473 GM taxa were obtained from MiBioGen consortium. CRS data (22,099 cases vs. 371,520 controls) were sourced from the FinnGen R12 cohort. Causal effects were estimated via Inverse Variance-Weighted (IVW), MR-Egger, weighted median, and Bayesian-weighted MR methods. Sensitivity analyses (heterogeneity and horizontal pleiotropy tests) were performed to validate robustness.

**Results:**

IVW analysis identified 20 GM taxa significantly associated with CRS risk (*p* < 0.05). Of these, 7 taxa (e.g., *Francisellales*, *Roseibacillus*, *Merdibacter massiliensis*) exhibited risk-increasing effects, while 13 taxa (e.g., *Firmicutes I*, *Succinivibrionaceae*) showed protective effects. Sensitivity analyses confirmed the absence of significant heterogeneity (Cochran’s *Q p* > 0.05) or pleiotropy (MR-Egger intercept *p* > 0.05). Bayesian-weighted MR validated 18 causal relationships (posterior probability > 95%), except for RUG420 sp900317985 and UBA7703 (non-significant).

**Conclusions:**

This MR study provides genetic evidence supporting causal roles of specific GM taxa in CRS pathogenesis. These findings highlight the gut-sinus axis as a potential therapeutic target and underscore the utility of large-scale biobanks (e.g., FinnGen) in advancing precision medicine.

**Level of evidence:**

Level 5. Mendelian Randomized (MR) studies are second only to randomized controlled trials in terms of the level of evidence.

## Highlights

• 20 gut microbes linked to sinusitis: 7 risk (Francisellales), 13 protective (Firmicutes I).
• Multi-method MR (IVW, Bayesian-weighted) and sensitivity tests confirm robustness.
• Gut-sinus axis highlighted as a novel therapeutic target for precision medicine.
• FinnGen cohort (393,619 individuals) supports large-scale causal inference.

## Introduction

Chronic Rhinosinusitis (CRS) is a prevalent inflammatory disease marked by persistent nasal and sinus mucosal inflammation, serving as a pathological foundation for various upper respiratory conditions. Symptoms such as nasal congestion and rhinorrhea significantly impact patients' quality of life. CRS arises from multiple factors that disrupt mucosal homeostasis and trigger abnormal inflammatory responses.^1^, ^2^, ^3^, ^4^, ^5^, ^6^, ^7^ Microbial dysbiosis, immune dysfunction, and impaired mucociliary clearance play key roles in CRS pathogenesis. Infections by bacteria and fungi can sustain inflammation through immune activation,^3^^,^^5^^,^^7^ while mucociliary dysfunction hinders secretion clearance, exacerbating inflammation.^3^^,^^7^ As inflammation progresses, mucosal swelling and polyp formation obstruct sinus ostia, impairing ventilation and drainage.^1^^,^^2^^,^^4^^,^^7^ Given its complexity and significant burden on patients and healthcare systems, a deeper understanding of CRS pathophysiology is essential for improving treatment strategies and patient outcomes.^4^^,^^6^, ^7^, ^8^

The Gut Microbiota (GM), a diverse microbial community in the human intestine, maintains a dynamic balance with the host and plays vital physiological roles.^9^, ^10^, ^11^, ^12^, ^13^, ^14^ Dysbiosis of GM has been linked to CRS,^15^, ^16^, ^17^, ^18^ yet its causal relationship remains unclear. Traditional case-control studies are often confounded by individual variations, environmental factors, and treatments, making it difficult to distinguish causation from correlation. While Randomized Controlled Trials (RCTs) are the gold standard for causal inference, their feasibility is limited by the need for large, heterogeneous populations, long study durations, complex interventions, and ethical constraints on invasive procedures. These challenges highlight the need for alternative approaches to explore the role of GM in CRS.

Mendelian Randomization (MR) is a powerful epidemiological tool that helps overcome key challenges in causal inference. By using Single Nucleotide Polymorphisms (SNPs) as Instrumental Variables (IVs), MR assesses causal relationships between exposures and outcomes while minimizing confounding and reverse causality, which are common in observational studies.^19^^,^^20^ Leveraging the random allocation of genes at conception, MR provides more reliable causal evidence than case-control and ecological studies, though it remains weaker than RCTs and their systematic reviews.^21^ Unlike RCTs, MR does not require large, heterogeneous cohorts, long follow-ups, or ethically restricted interventions. By analyzing genetic data, MR enables precise investigation of the causal links between specific gut microbes and CRS, offering novel insights into CRS pathogenesis. It has been widely applied in studying the gut microbiota’s role in metabolic and autoimmune diseases.^22^^,^^23^

## Methods

### Data sources

The genome-wide association study (GWAS) data for the GM were obtained from the European Bioinformatics Institute’s GWAS database, accessible at https://www.ebi.ac.uk/gwas/. This database provides detailed records of the characteristics of various GM taxa. A total of 473 taxa were meticulously selected for analysis in this study, covering 10 phyla, 18 classes, 24 orders, 58 families, 143 genera, 213 species, and 7 unclassified microbial groups. The GWAS summary data for CRS (GWAS ID: finn-b-J10_CHRONSINUSITIS) were sourced from the Finnish database, R12 release, with access available through the Finnish Genomics Research official website at https://www.finngen.fi/en. This dataset includes individuals of European ancestry, with the research sample consisting of 22,099 CRS cases and 371,520 controls (Table 1). All summary data used in this study were obtained from publicly available databases, ensuring full legal and ethical compliance regarding data usage. The relevant ethical approval and informed consent from patients were obtained in the original study.

**Table 1:** GWAS information.

Variable	Year	Sample size	Population	Gender
Gut microbiota	2022	5959	European	Mixed gender
Chronic sinusitis	2024	393,619	European	Mixed gender

### Selection of instrumental variables

In this study, rigorous selection and evaluation of instrumental variables were performed to investigate the causal relationship between GM and CRS. At the beginning of the analysis, we set strict criteria based on the European genomic sample data. The threshold for genome-wide statistical significance was set at *p* < 1 × 10^−5^ (due to the limited number of SNPs with *p* < 5 × 10^−8^, the p-value threshold was moderately increased). Furthermore, parameters for linkage disequilibrium analysis were set (κb = 10,000,  *r*^2^ < 0.001), and SNPs with F-values smaller than 10 were excluded to reduce the influence of weak instrumental variables. The calculation formula for the F-value is as follows: F = [(N - κ - 1) / κ] × r2 / (1 - r2).Where r^2^ is calculated by the formula: r2 = 2 × β2 × EAF × (1 - EAF) / 2 × β2 × EAF × (1 - EAF) + SE2 × 2 × N × EAF × (1 - EAF),where EAF is the effect allele frequency, β is the allele effect size, SE is the standard error, N is the sample size, and k is the degrees of freedom (equal to 1). *R* version 4.4.2 was used for analysis with the Two Sample MR package (version 0.6.8).

### Mendelian randomization analysis

**Fig. 1:**
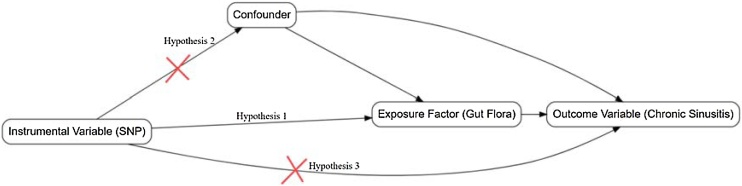
Core assumptions of mendelian randomization.

We employed the two-sample Mendelian Randomization (MR) method to assess the causal effect of GM on CRS. The MR analysis relies on three core assumptions: 1) Instrumental variable relevance assumption: The SNPs used as instrumental variables must be strongly associated with the exposure factor. 2) Independence assumption: Genetic variations should be independent of confounding factors that affect both the exposure and the outcome. 3) Exclusion assumption: SNPs must affect the outcome only through the exposure factor, without influencing the outcome through other pathways. The validity of these three assumptions is crucial to ensure the reliability of the MR analysis results^24^ (Fig. 1).

Five methods were employed to assess the causal relationship between the GM and CRS: Inverse Variance Weighted (IVW), MR-Egger regression, weighted median, simple model, and weighted mode. IVW is a common approach in MR analysis that combines effect estimates of multiple instrumental variables using inverse variance weighting, providing more precise causal effect estimates.

For data reliability analysis, Cochran’s *Q* test assessed heterogeneity among instrumental variables, with *p* < 0.05 indicating significant heterogeneity, necessitating a random or fixed effects model. If heterogeneity was detected, MR-Egger regression evaluated horizontal pleiotropy, considered present if *p* < 0.05. The MR-PRESSO method further corrected pleiotropy by identifying and removing outliers. Leave-one-out analysis tested data stability by assessing whether excluding a single SNP altered causal estimates. Together, these methods ensured a comprehensive reliability assessment. Additionally, Bayesian Weighted Mendelian Randomization (BWMR) was employed to mitigate biases from weak instruments and pleiotropy, enhancing result stability and robustness through an adaptive Bayesian framework.^25^

## Results

### Mendelian randomization results

**Fig. 2:**
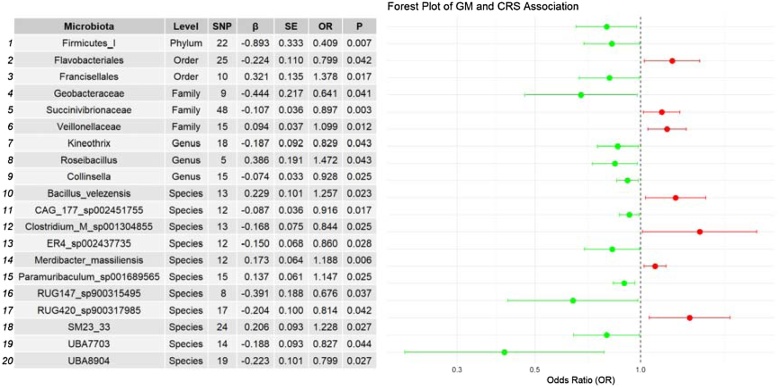
Gut microbiota and chronic rhinosinusitis: MR analysis. SNP, represents the number of Single Nucleotide Polymorphisms; β, is the effect size of the allele; SE, is the Standard Error of β.

**Fig. 3:**
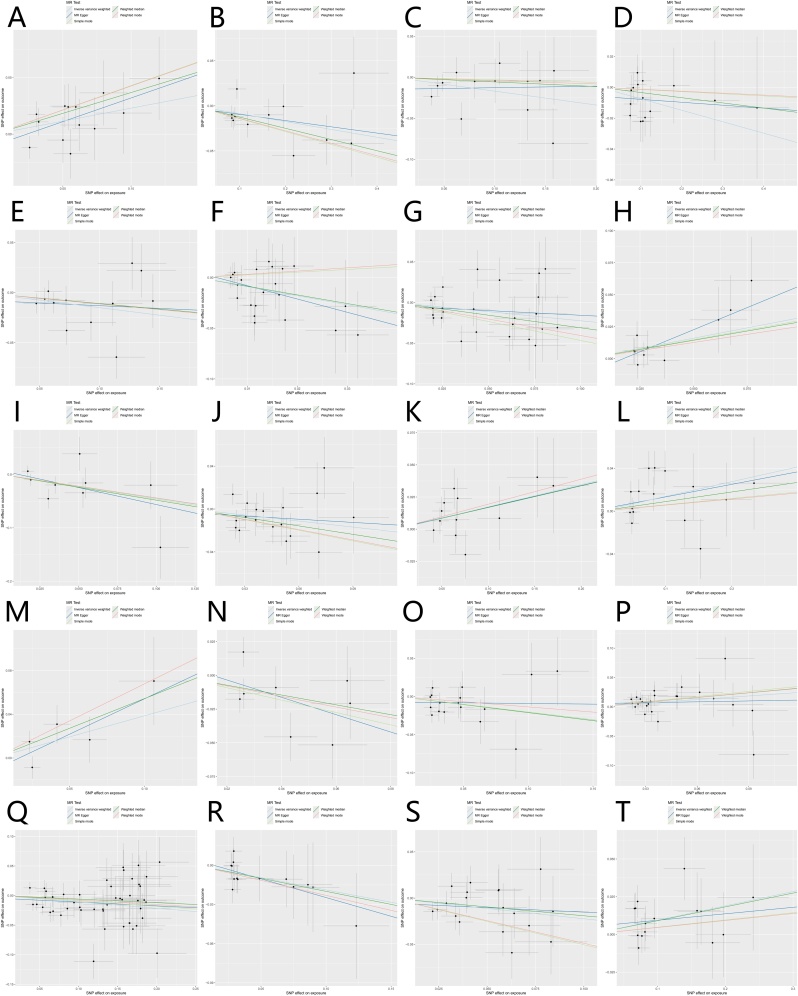
(A‒T) Scatter plot of the causal effect of GM on CRS using MR analysis. (A) *Bacillus velezensis*; (B) *CAG-177 sp002451755*; (C) *Clostridium M sp001304855*; (D) *Collinsella*; (E) *ER4 sp002437735*; (F) *Firmicutes I*; (G) *Flavobacteriales*; (H) *Francisellales*; (I) *Geobacteraceae*; (J) *Kineothrix*; (K) *Merdibacter massiliensis*; (L) *Paramuribaculum sp001689565*; (M) *Roseibacillus*; (N) *RUG147 sp900315495*; (O) *RUG420 sp900317985*; (P) *SM23-33*; (Q) *Succinivibrionaceae*; (R) *UBA7703*; (S) *UBA8904*;(T) *Veillonellaceae.*

**Fig. 4:**
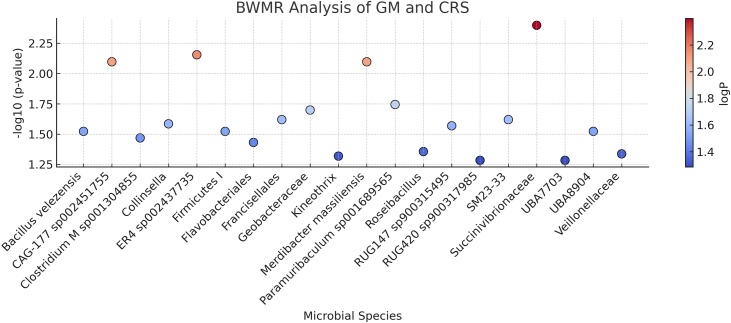
Volcano plot of Bayesian Weighted MR (BWMR) analysis for GM and CRS.

IVW analysis revealed 20 GM taxa significantly associated with CRS (*p* < 0.05), involving 326 SNPs. These results from the Mendelian randomization analysis of 20 GM taxa with significant causal relationships to CRS are summarized in Fig. 2. The results of the IVW analysis showed that 7 GM taxa were positively associated with CRS, and 13 GM taxa were negatively associated with CRS. The following GM taxa showed a positive correlation with CRS: *Francisellales* (OR = 1.378, 95% CI 1.057–1.797, *p* = 0.017), *Roseibacillus* (OR = 1.472, 95% CI 1.012–2.141, *p* = 0.043), *Merdibacter massiliensis* (OR = 1.188, 95% CI 1.048–1.348, *p* = 0.006), *Paramuribaculum sp001689565* (OR = 1.147, 95% CI 1.016–1.295, *p* = 0.025), SM23-33 group (OR = 1.228, 95% CI 1.023–1.475, *p* = 0.027), *Veillonellaceae* (OR = 1.099, 95% CI 1.020–1.183, *p* = 0.012), and *Bacillus velezensis* (OR = 1.257, 95% CI 1.031–1.533, *p* = 0.023). On the other hand, the following GM taxa showed a negative correlation with CRS: *Firmicutes I* (OR = 0.409, 95% CI 0.212–0.787, *p* = 0.007), *Flavobacteriales* (OR = 0.799, 95% CI 0.643–0.992, *p* = 0.042), *Geobacteraceae* (OR = 0.641, 95% CI 0.418–0.982, *p* = 0.041), *Succinivibrionaceae* (OR = 0.897, 95% CI 0.835–0.965, *p* = 0.003), *Kineothrix* (OR = 0.829, 95% CI 0.691–0.994, *p* = 0.043), *Collinsella* (OR = 0.928, 95% CI 0.869–0.990, *p* = 0.025), *CAG-177 sp002451755* (OR = 0.916, 95% CI 0.852–0.985, *p* = 0.017), *Clostridium M sp001304855* (OR = 0.844, 95% CI 0.728–0.979, *p* = 0.025), *ER4 sp002437735* (OR = 0.860, 95% CI 0.751–0.984, *p* = 0.028), *RUG147 sp900315495* (OR = 0.676, 95% CI 0.467–0.978, *p* = 0.037), *RUG420 sp900317985* (OR = 0.814, 95% CI 0.668–0.993, *p* = 0.042), *UBA7703* group (OR = 0.827, 95% CI 0.688–0.995, *p* = 0.044), *UBA8904* group (OR = 0.799, 95% CI 0.655–0.975, *p* = 0.027), (Fig. 3). Bayesian weighted Mendelian randomization (BWMR) analysis also confirmed the association between 20 GM taxa and CRS, with p-values for *RUG420 sp900317985* and the *UBA7703* group greater than 0.05, while the remaining 18 associations showed varying degrees of statistical significance (Table 2, Fig. 4).

**Table 2:** The results of Mendelian randomization analysis using the BWMR method for GM and CRS.

Microbiota	Method	Beta	Or	*Or_lci95*	*Or_uci95*	pval
*Bacillus velezensis*	BWMR	0.238	1.269	1.024	1.574	0.030
*CAG-177 sp002451755*	BWMR	−0.091	0.913	0.853	0.977	0.008
*Clostridium M sp001304855*	BWMR	−0.173	0.841	0.717	0.987	0.034
*Collinsella*	BWMR	−0.078	0.925	0.863	0.991	0.026
*ER4 sp002437735*	BWMR	−0.163	0.849	0.754	0.957	0.007
*Firmicutes I*	BWMR	−0.823	0.439	0.209	0.921	0.030
*Flavobacteriales*	BWMR	−0.234	0.791	0.635	0.986	0.037
*Francisellales*	BWMR	0.321	1.378	1.043	1.822	0.024
*Geobacteraceae*	BWMR	−0.478	0.620	0.415	0.927	0.020
*Kineothrix*	BWMR	−0.198	0.820	0.674	0.998	0.048
*Merdibacter massiliensis*	BWMR	0.180	1.197	1.047	1.368	0.008
*Paramuribaculum sp001689565*	BWMR	0.148	1.160	1.026	1.312	0.018
*Roseibacillus*	BWMR	0.427	1.532	1.011	2.323	0.044
*RUG147 sp900315495*	BWMR	−0.435	0.647	0.440	0.953	0.027
*RUG420 sp900317985*	BWMR	−0.217	0.805	0.646	1.002	0.052
*SM23-33*	BWMR	0.222	1.248	1.029	1.513	0.024
*Succinivibrionaceae*	BWMR	−0.112	0.894	0.829	0.964	0.004
*UBA7703*	BWMR	−0.191	0.826	0.682	1.002	0.052
*UBA8904*	BWMR	−0.233	0.792	0.642	0.977	0.030
*Veillonellaceae*	BWMR	0.081	1.084	1.001	1.174	0.046

### Sensitivity analysis

**Fig. 5:**
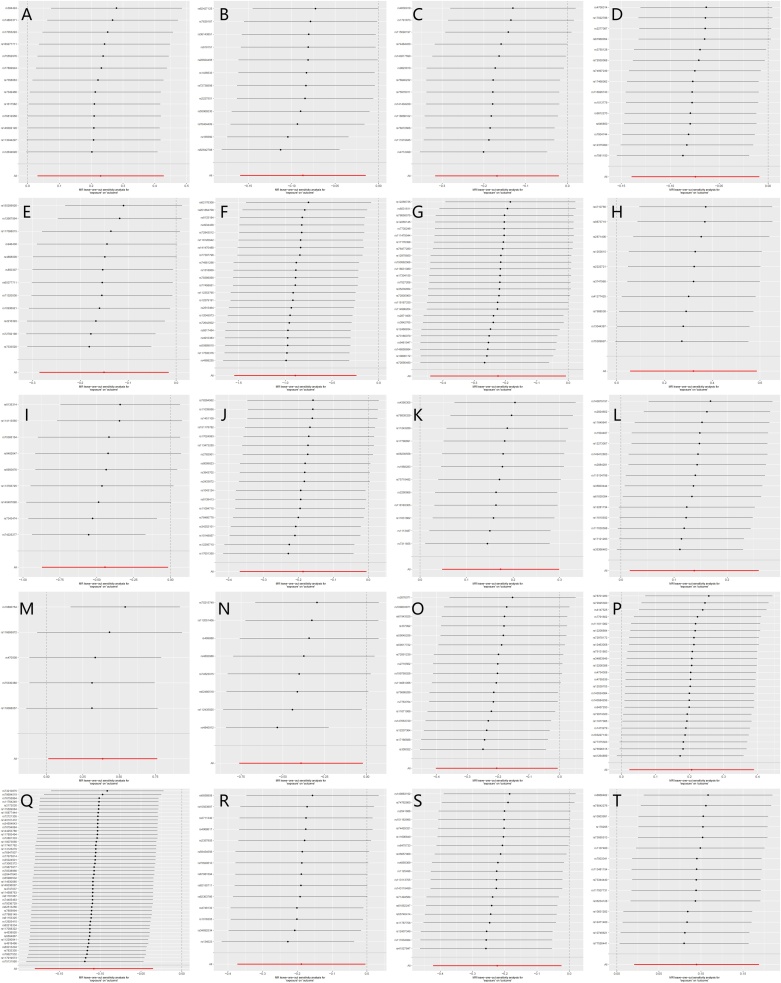
(A‒T) Leave-one-out plot of MR analysis on the causal effect of GM on CRS. (A) *Bacillus velezensis*; (B) *CAG-177 sp002451755*; (C) *Clostridium M sp001304855*; (D) *Collinsella*; (E) *ER4 sp002437735*; (F) *Firmicutes I*; (G) *Flavobacteriales*; (H) *Francisellales*; (I) *Geobacteraceae*; (J) *Kineothrix*; (K) *Merdibacter massiliensis*; (L) *Paramuribaculum sp001689565*; (M) *Roseibacillus*; (N) *RUG147 sp900315495*; (O) *RUG420 sp900317985*; (P) *SM23-33*; (Q) *Succinivibrionaceae*; (R) *UBA7703*; (S) *UBA8904*; (T) *Veillonellaceae*.

To assess the robustness of the model, sensitivity analyses were conducted (Table 3). The heterogeneity test showed no significant heterogeneity in MR-Egger and IVW methods, indicating high consistency of instrumental variables and strengthening causal inference. The horizontal pleiotropy test detected no significant pleiotropic effects, confirming that instrumental variables were not influenced by alternative pathways. Additionally, MR-PRESSO analysis identified no outliers, suggesting minimal impact from abnormal instrumental variables. The leave-one-out analysis demonstrated that removing any single SNP did not alter the causal effect estimates, indicating result stability (Fig. 5). Overall, these findings support the reliability of the causal estimates, unaffected by heterogeneity, pleiotropy, or outliers.

**Table 3:** Sensitivity analysis results.

Level	Microbiota	Heterogeneity test		Horizontal pleiotropy test	
		MR Egger (Q_pval)	Inverse variance weighted (Q_pval)	Egger_intercept	Egger_intercept pval
Phylum	*Firmicutes I*	0.587	0.630	0.005	0.598
Order	*Flavobacteriales*	0.190	0.218	−0.004	0.593
Order	*Francisellales*	0.853	0.820	−0.011	0.321
Family	*Geobacteraceae*	0.129	0.168	0.007	0.630
Family	*Succinivibrionaceae*	0.232	0.249	−0.004	0.508
Family	*Veillonellaceae*	0.628	0.679	0.005	0.600
Genus	*Kineothrix*	0.488	0.549	−0.003	0.714
Genus	*Roseibacillus*	0.241	0.306	−0.010	0.551
Genus	*Collinsella*	0.753	0.792	−0.005	0.579
Species	*Bacillus velezensis*	0.711	0.725	−0.009	0.415
Species	*CAG-177 sp002451755*	0.195	0.256	−0.002	0.822
Species	*Clostridium M sp001304855*	0.459	0.409	−0.014	0.223
Species	*ER4 sp002437735*	0.102	0.130	−0.007	0.635
Species	*Merdibacter massiliensis*	0.493	0.583	3.5E-4	0.975
Species	*Paramuribaculum sp001689565*	0.083	0.113	0.001	0.881
Species	*RUG147 sp900315495*	0.085	0.117	0.009	0.649
Species	*RUG420 sp900317985*	0.435	0.458	−0.007	0.423
Species	*SM23-33*	0.420	0.454	0.005	0.525
Species	*UBA7703*	0.932	0.951	0.003	0.654
Species	*UBA8904*	0.541	0.590	−0.005	0.608

## Discussion

Currently, most studies have focused on the effects of eosinophils and certain immune memory cells on CRS.^26^, ^27^, ^28^ With the increasing understanding of CRS pathogenesis, GM has gradually garnered attention from researchers. GM, as a complex and dynamic ecosystem in the human body, plays a crucial role in maintaining host health and influencing the development of various diseases.^29^^,^^30^ This study utilized MR methods and GWAS data to explore the causal relationships between CRS and 473 types of GM. Our findings identified 20 GM species that are causally associated with CRS. This result provides a new direction for studying the pathogenesis of CRS and offers new biomarkers for precision medicine, which has significant implications for clinical practice and future research.

Among the Gut Microbiota (GM) with a positive causal relationship with Chronic Rhinosinusitis (CRS), *Francisellales*, *Roseibacillus*, and *Merdibacter massiliensis* are particularly noteworthy, aligning with previous findings.^31^ *Francisellales* may influence CRS onset by modulating the immune system, triggering the release of inflammatory cytokines such as interleukins and tumor necrosis factor. These mediators can enter the bloodstream, reach the nasal and sinus mucosa, activate immune cells, and exacerbate local inflammation. *Roseibacillus* may impair gut barrier function by altering the intestinal microenvironment, allowing harmful substances to enter circulation, thereby disrupting nasal and sinus metabolism and immune functions, ultimately promoting CRS development.^7^^,^^32^ *Merdibacter massiliensis*, an anaerobic Gram-negative bacterium primarily isolated from the human ileum, lacks catalase and oxidase activity, suggesting its adaptation to the low-oxygen gut environment. While it contributes to metabolic homeostasis, bioinformatic analyses suggest its potential pathogenicity.^33^

Among the GM with a negative causal relationship with CRS, *Firmicutes I* and *Succinivibrionaceae* are particularly significant. *Firmicutes I*, a major bacterial phylum in the human gut, plays a crucial role in immune homeostasis. It is known to harbor genes responsible for fermenting dietary fiber, thereby promoting the secretion of anti-inflammatory cytokines such as interleukin-10 while suppressing pro-inflammatory cytokines. This modulation of immune responses may reduce systemic inflammation and alleviate mucosal inflammation in the nasal and sinus tissues. Additionally, *Firmicutes I* enhances gut barrier function, preventing harmful microorganisms and their metabolic byproducts from entering the bloodstream, which in turn reduces CRS susceptibility.^34^^,^^35^ *Succinivibrionaceae*, primarily found in the rumen, is known for optimizing microbial structure, promoting feed digestion, and exhibiting probiotic effects. Studies suggest that *Succinivibrionaceae* modulates gut microbiota composition, stabilizes metabolic processes, and reduces inflammation. Notably, research on the tammar wallaby microbiota has revealed that *Succinivibrionaceae* (WG-1) is associated with low methane emissions, indicating its potential role in metabolic regulation. This finding highlights a possible parallel in the human gut, where specific microbial strains may influence host metabolism and immune function, contributing to CRS prevention.^36^, ^37^, ^38^

These findings suggest that certain GM species may serve as potential therapeutic targets for CRS. While some microbiotas have been established as causally linked to CRS, the precise mechanisms underlying their effects remain incompletely understood. Future studies should focus on elucidating the immunoregulatory and metabolic pathways involved, paving the way for novel microbiome-based therapeutic strategies for CRS.

The strength of this study lies in its use of the large-scale Finnish health database and GWAS data, employing the MR method to effectively control confounding factors and enhance the reliability and accuracy of the results. The Finnish database provided high-quality genetic data, enabling more precise identification of the potential causal relationships between microbiota and diseases. This provides scientific evidence for the early prediction and individualized treatment of CRS. The insights from this study can serve as a reference for establishing large health databases globally and encourage the integration of multi-center medical data to support the development of precision medicine. However, there are some limitations in this study. First, the study population predominantly consisted of individuals of European ancestry, which may limit the generalizability of the findings, as the relationship between GM and CRS may differ in other racial groups. Second, although this study identified causal relationships, MR research is inherently based on statistical inference and cannot directly prove causality. Further animal experiments or clinical trials are necessary to confirm whether these gut microbiotas directly influence CRS development. Additionally, future research could combine multi-omics data, such as metagenomics and metabolomics, to further explore the mechanisms by which GM influences CRS pathogenesis and validate the robustness of the findings.

## Conclusion

This study reveals multiple causal relationships between gut microbiota and CRS, providing new insights into the pathogenesis of CRS. As more research is conducted, modulating the gut microbiota could potentially become a novel therapeutic strategy for CRS, offering new possibilities for the future development of precision medicine.

## ORCID IDs

Chenguang Zhang: 0009-0005-0660-3763, Yicong Wang: 0009-0002-3950-775X, Chenghao Hu: 0000-0002-4313-686X, Bin Guo: 0000-0002-6490-8339, Huwei Jiang: 0009-0001-1029-6832, Chunlong Zhao: 0009-0007-9836-4812, Yuwen Wang: 0009-0001-5415-7180

## Funding

The study was supported by the 2021 Qinghai Kunlun Elite High-end Innovation and Entrepreneurship Talent Program (Grant nº 2021-13).

## Declaration of competing interest

The authors declare no conflicts of interest.
